# IMPACT OF A STROKE CARE PATHWAY ON SELF-PERCEIVED HEALTH 3 MONTHS AFTER STROKE

**DOI:** 10.2340/jrm.v57.42443

**Published:** 2025-02-18

**Authors:** Elin BERGH, Torunn ASKIM, Ole Morten RØNNING, Stian LYDERSEN, Bente THOMMESSEN

**Affiliations:** 1 Department of Neuromedicine and Movement Sciences, NTNU, Trondheim; 2 Department of Neurology, Akershus University Hospital, Akershus; 3 Regional Centre for Child and Youth – Mental health and Child Welfare (RKBU) Department of Mental Health NTNU, Trondheim, Norway

**Keywords:** stroke, care pathway, time-delay, self-perceived health, rehabilitation

## Abstract

**Background:**

A national stroke care pathway aiming at reducing time delays in stroke care was implemented in Norway in 2018. This study aimed to investigate the impact of goal attainment in the pre- and intrahospital care pathway on self-perceived health 3 months after stroke.

**Methods:**

Data from the Norwegian Stroke Register (NSR) from 2019 were used. Patients were classified into attainment or non-attainment groups, depending on goal achievement of the SCP. Linear regression analyses were used to assess a possible association between goal attainment and self-perceived health evaluated by EQ-5D-5L and EQ-VAS at 3 months post-stroke.

**Results:**

Among 4,133 included patients, 908 (22%) were included in the attainment group. We found no effect of goal attainment upon mean EQ-5D-5L. However, when adjusting for stroke severity, attaining the SCP was significantly associated with self-perceived health.

**Conclusion:**

In this study, with a cohort of patients with mild to moderate strokes, we found no association between attaining goals of the stroke care pathway and self-perceived health. However, stroke severity might have acted as a suppressor variable underscoring the importance of stroke severity for self-perceived health.

## LAY ABSTRACT

Stroke is a major cause of disability and death. Researchers are working to improve stroke treatment and reduce its impact on patients. In our study, we looked at whether a care plan designed to treat patients faster and reduce delays to treatment and rehabilitation would improve their self-rated health 3 months after stroke. Using data from the Norwegian Stroke Register, we found no difference in self-rated health between patients who met the care plan goals and those who did not. Instead, stroke severity was the main factor affecting patients’ health perception. This indicates a need to explore alternative approaches to enhance self-perceived health after stroke.

Despite a significant reduction in age-standardized deaths from 1990 to 2021, stroke is still the third leading cause of death and the fourth most common cause of Disability Adjusted Life Years (DALYs) globally (1). The evidence for stroke unit treatment encompassing thrombolysis and thrombectomy represents great breakthroughs in acute management over the past decades (2–4). Hence, international guidelines recommend that patients with a possible stroke should be immediately transported to the closest hospital capable of providing emergency stroke care, including intravenous (i.v.) thrombolysis (5). Clinical pathways have been established to promote organized and efficient patient care based on the best evidence and guidelines; however, research focusing on in-hospital care pathways for stroke is limited and so far shows no effect on patient-related outcomes (6–8).

In Norway, approximately 10,000 patients are hospitalized with acute stroke each year (9). Despite the well-established National guidelines, treatment disparities between different regions and hospitals were observed (10). To reduce these differences, the Norwegian healthcare authorities introduced a national stroke care pathway (SCP) aiming to optimize stroke care and to promote uniform treatment and rehabilitation across the country by reducing time delays in the assessment, diagnostics, treatment, and rehabilitation of stroke (11).

The pathway is organized in 2 phases aligned with key national quality indicators. The time-specific goals of phase one, which include the pre- and intrahospital pathway, are displayed in Table I.

**Table I:** The standardized pathway of stroke care

Point of measurement	Goal
1. Time for symptom onset	Registration
**2A. Time from onset to contact with the EMS**	≤15 min
2B. Time from contact with ambulance to arrival of ambulance	≤25 min
2C. Time from arrival to departure of ambulance	≤25 min
**3. Time from symptom onset to hospitalization**	≤4 h
**4. Time from hospital admission to CT/MRI**	≤15 min
5. Time from hospital admission to thrombolysis	≤40 min
6A. Thrombectomy	Registration
6B. Thrombectomy	Directly admitted patients: puncture ≤60 min Transferred patients: puncture ≤40 min
6C. Thrombectomy: time for recanalization	Registration
**7. Time from hospital to stroke unit admission**	≤3 hours
8A. Time from admission to examination of ICA	≤3 days
8B. Time to carotid endarterectomy	≤14 days
9. Interdisciplinary assessment in a stroke unit	≤7 days
10. Time from discharge until rehabilitation	Registration

The bold text indicate the goals that are analysed in our study.

EMS: emergency medical service; CT: computed tomography; MRI: magnetic resonance image; ICA: internal carotid artery.

Much of the existing research focuses on functional outcomes after stroke and less is known about how patients themselves view their recovery, and which factors influence their perception of health.

Patient-reported outcome measures (PROMs) capture additional health aspects beyond the clinician-reported measures and may not correlate well with the Modified Rankin Scale (mRS) (12–14). Their ability to independently predict future outcomes such as mortality and hospitalization emphasize their importance in clinical studies (15).

Self-perceived health (SPH) refers to the perception of a person’s health in general. Several studies show that individuals report reduced SPH after stroke compared with the general population and the differences are found to be larger in the domains of physical health than those of mental health (16). Stroke severity, comorbidity, age, and educational level are individual factors found to be associated with SPH (17).

In two previous studies we found a positive association between goal attainment of the stroke care pathway and independence 3 months post -stroke, but observed no overall effect when comparing outcomes before and after implementation (18)^1^. It would therefore be of great interest to investigate whether significant delays in the time-specific treatment goals of the pathway also have an impact on the patient’s self-perceived health.

Hence, the aim of the present study was to investigate the impact of goal-attainment in the pre- and intrahospital care pathway on self-perceived health 3 months after stroke. We hypothesized that we would find a positive association between attainment of the most important goals of the stroke care pathway and improved self-perceived health 3 months post-stroke.

## Methods

This study utilized data from the Norwegian Stroke Registry (NSR), which includes medical information on all patients admitted to Norwegian hospitals with acute stroke in 2019, 1 year after the introduction of the SCP. We have previously described the register, which has a coverage rate of 87%. The quality has been confirmed at level 4, the highest attainable ranking (19).

In the present study, all patients registered in the NSR with 3 months’ follow-up and required data were included.

### Data collection

The NSR is a national, compulsory register and all 50 acute care hospitals in Norway are required to submit medical information for all patients hospitalized with acute stroke. Data collection takes part when the patient is in hospital, either by attending practitioners or by dedicated nurses. Data assessment 3 months post-stroke is carried out by healthcare personnel at the outpatient clinic, over the phone, or through correspondence, where patients complete and return pre-sent forms.

Data retrieved from the NSR comprise: baseline characteristics (age, sex, marital status, residence, health region), cardiovascular risk factors (hypertension, atrial fibrillation, previous transient ischaemic attack [TIA], previous stroke, diabetes mellitus [DM]), neurological function at admission and 24 h after admission measured by the National Institutes of Health Stroke Scale (NIHSS), functional status pre-stroke and 3 months post-stroke evaluated by mRS, registered target times of the stroke care pathway (time of onset of symptoms, time of contact with the Emergency Medical Services (EMS), time of hospitalization, time of imaging, time of thrombolysis, time of admission to a stroke unit and treatment received (thrombolysis yes vs no). SPH 3 months post-stroke was assessed by EQ5D-5L and EQ-VAS (20, 21).

### Assessment of stroke care pathway attainment

In the present study, we evaluate the part of the stroke care pathway that covers the period from stroke onset until the patient is ready to be discharged from hospital (see Table I). This part of the pathway includes 3 distinct categories of goals: goals serving solely for registration purposes (goal 1, 6A, 6C, and 10), goals with target times applicable to subgroups of patients undergoing i.v. thrombolysis (6.3%) or endarterectomy (3.3%), (goal 5, 6B, and 8B) and finally goals with target times that apply to the entire stroke population (goal 2, 3, 4, 7, 8A, and 9) (see Table I). In this study, our primary focus was on the last category of goals due to their known importance regarding stroke outcome and that they were applicable to the whole stroke population.

Goals 8A and 9 were excluded due to missing data. The target times regarding “time from contact with EMS until ambulance arrival” and “time from arrival until departure of the ambulance” (2B and 2C) are largely encompassed by the broader “time from onset of symptoms until hospital arrival” (goal 3).

Considering this, the included goals of the stroke care pathway were:

(2). Time from symptom onset to contact with the EMS ≤15 mins.
(3). Time from symptom onset to admission to hospital ≤4 h.
(4). Time from admission to hospital to radiological examination ≤15 min.
(7). Time from arrival in hospital to admission to a stroke unit ≤3 h.

“Attainment” was defined as fulfilling at least 3 of the 4 selected SCP goals within the recommended target times. “Non-attainment” was defined as fulfilling 0–2 of these goals.

It is crucial to highlight that the goals we included in this study were determined before we conducted the analyses (4).

### Self-perceived health assessment

We used the EQ-5D-5L score to evaluate SPH, with its suitability and psychometric validity for stroke patients already established (21, 22). The scale consists of 2 parts. The first part assesses the health in 5 dimensions (5D): (*i*) Mobility, (*ii*) Self-care, (*iii*) Usual activities, (*iv*) Pain/discomfort, and (*v*) Anxiety/depression. Each dimension has 5 levels of response (5 L) from 1p: “no problems” to 5p: “extreme problems”. By utilizing a formula that assigns numerical values to each level within every dimension, each health state can be converted into an index score (22). The EQ-5D-5L Index Value Calculator Version 2.0, developed by the EuroQol Group, was used for this conversion. We used the value set from Denmark as no validated Norwegian value set exists (23). The index scores range from +1 to −0.624, where 1 represents the best possible health, 0 indicates death, and scores <0 signify a health condition worse than death.

The EQ-VAS is a visual analogue scale on which the participants rate their perceived health status on a scale from 0 to 100, where 0 points indicate “the worst health condition you can imagine” and 100 points “the best health condition you can imagine” (24). The construct validity of the EQ-VAS is generally considered satisfactory (25).

Registration of the EQ-5D-5L and EQ-VAS was performed at the outpatient clinic or by phone interview 3 months post-stroke.

### Statistics

Descriptive statistics are presented as counts and frequencies for categorical variables and means and standard deviations (SD) for continuous variables. Groups were compared using the Pearson χ^2^ test for categorical variables and the *t*-test for continuous variables.

To examine the impact of goal attainment on SPH, linear regression analyses were performed with EQ-5D-5L or EQ-VAS as dependent variable, and attainment vs non-attainment as main covariate. The analyses were performed unadjusted and adjusted for age, sex, living alone pre-stroke, hypertension, previous stroke, previous TIA, atrial fibrillation (AF), diabetes mellitus (DM), NIHSS on admission, and pre-stroke mRS (0–2 vs 3–5). Normality of residuals was checked by visual inspection of Q–Q plots.

Subsequently, regression-based mediation analysis was conducted to explore whether thrombolysis acts as a mediating variable between goal attainment (yes vs no) and SPH. The mediation analysis was performed independent of an existing predetermined statistically significant association of goal attainment on self-perceived health, and it was adjusted for age, gender, and NIHSS on admission.

The main analyses were conducted using SPSS (IBM SPSS Statistics for Windows, version 29.0: IBM Corp, Armonk, NY, USA). The mediation analysis was performed using the “mediate” function in Stata version 18.0 (StataCorp LLC, College Station, TX, USA).

## Results

**Fig. 1:**
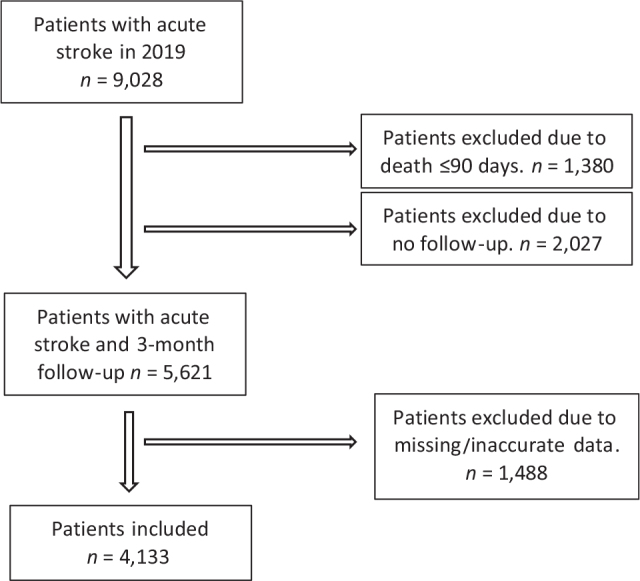
Flowchart showing inclusion/exclusion of patients and missing data.

In total, 9,028 patients with acute stroke were registered in the NSR in 2019 and of these 4,133 patients (46 %) were included in our study. The main reasons for exclusion were no follow-up assessment, missing measures on SCP attainment and SPH at 3 months, and death within 3 months as shown in Fig. 1.

### Demographic data

Table II presents goal attainment and Table III the patient characteristics and clinical data for the 2 groups. Patients in the attainment group were non-significant younger (mean 71.1 vs 72.4 years, *p* = 0.23) and were to a lesser degree living alone pre-stroke (32.2% vs 40.2%, *p* < 0.001). More than 5 times as many patients in the attainment group received thrombolysis compared with those in the non-attainment group (60% vs 11%, *p* < 0.001). Further, the attainment group had a higher pre-stroke independence rate (mRS 0–2: 95.7% vs 93.6%, *p* = 0.023) and experienced more severe stroke as indicated by a higher NIHSS score on admission (5.3 vs 3.3, *p* < 0.001).

**Table II:** Proportion of goal attainment in the 2 groups

SCP goals	Attainment *n* (%)	Non-attainment *n* (%)
Symptom-onset to contact with EMS, n%	428 (47)	80 (2.5)
Symptom-onset to admission in hospital, n%	908 (100)	953 (30)
Admission in hospital to CT/MRI, n%	757 (83)	417 (13)
Admission in hospital to admission in stroke unit, n%	846 (93)	1633 (51)

EMS: emergency medical services; CT: computer tomography; MRI: magnetic resonance imaging.

**Table III:** Patient characteristics in total and stratified by attainment of 3 or 4 goals in the stroke care pathway

	Total *n* = 4,133	Total attainment	Attainment	Total non-attainment	Non-attainment
Demographics					
Age, mean (SD), y	4,133	908	71.1 (12.7)	3,225	72.4 (12.3)
Female sex, *n* (%)	4,133	908	392(43.2)	3,225	1395 (43.3)
Living alone pre-stroke	4,108	900	290 (31.9)	3,208	1291 (40.2)
Risk factors					
Hypertension	4,114	907	511 (56.3)	3,207	1810 (56.4)
Diabetes mellitus	4,126	906	129 (14.2)	3,220	606 (18.8)
Previous TIA	4,086	900	94 (10.4)	3,186	299 (9.4)
Previous stroke	4,117	904	187 (20.7)	3,213	642 (20.0)
Previous atrial fibrillation	4,118	905	180 (19.9)	3213	686 (21.4)
Ischaemic stroke	4,133	908	821 (90.4)	3,225	2965 (91.4)
Thrombolysis	4,130	907	546 (60.2)	3,223	351 (10.9)
Degree of disability					
mRS pre-stroke - mRS 0–2 - mRS 3–5	3,787	860	823 (95.7) 37 (4.3)	2,927	2740 (93.6) 187 (6.4)
mRS 3 months - mRS 0–2 - mRS 3–5	4,133	908	718 (79.1) 190 (20.9)	3,225	2560 (79.4) 665 (20.6)
NIHSS at admission, mean, (SD)	3,610	884	5.3 (5.3)	2,726	3.3 (4.1)
NIHSS at 24h, mean (SD)	3,198	768	3.1 (4.0)	2,430	2.5 (3.2)

SD: standard deviation; TIA: transient ischaemic attack; mRS: modified Rankin scale.

All measures are given as *n* (%).

By comparing the included patients with the whole population, we found that there were substantial differences in NIHSS on admission (NIHSS 3.8 vs 5.7) and at 24 h (2.6 vs 4.3). Those who died within 3 months (1,285 patients) had NIHSS 13.5 on admission and 13.0 at 24 h.

### Self-perceived health

**Fig. 2:**
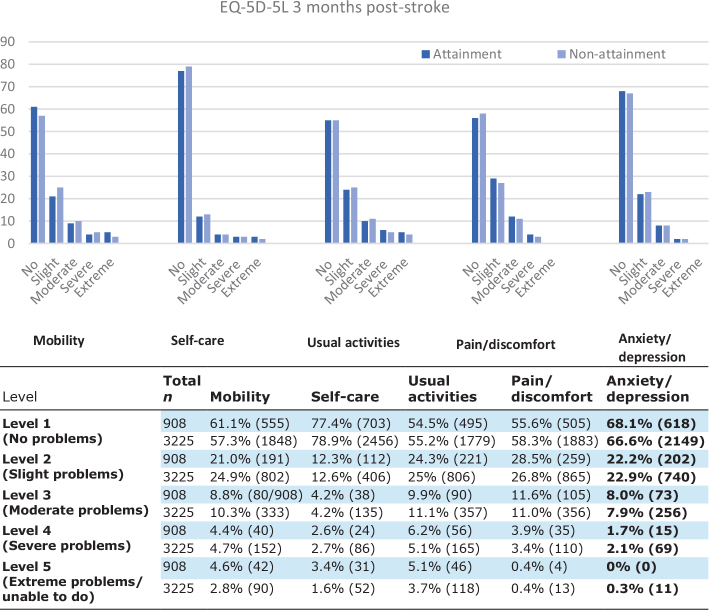
(A) Distribution of the EQ-5D-5L dimensions based on 5 degrees of problems 3 months post-stroke. (B) Numbers explaining the figure. The upper, blue columns show achievement group numbers and the lower, white the non-achievement group.

Fig. 2 illustrates the distribution of the 5 different dimensions of the EQ-5D-5L assessed at 3 months post-stroke for the attainment and non-attainment groups. As illustrated, a vast majority of patients in both groups report no or minor problems in all 5 dimensions.

The mean EQindex in the attainment group was 0.80 and in the non-attainment group. 0.79. The mean EQ-VAS was 69 in both groups. The results of the regression analysis are presented in Table IV.

**Table IV:** Simple and multiple linear regression with EQ-5D-5L and EQ-VAS as dependent variables and attainment of care pathway as main covariate

		EQ-5D-5L index			EQ-VAS	

Covariate	Regression coefficient	CI	*p*-value	Regression coefficient	CI	*p*-value
Attainment of SCP unadjusted	0.009	–0.024 to 0.006	0.231	0.012	–1.577 to 1.601	0.988
Adjusted separately for						
• Female sex	0.012	–0.27 to 0.003	0.112	0.219	–1.362 to 1.801	0.786
• Age	0.013	–0.028 to 0.01	0.071	0.333	–1.242 to 1.908	0.678
• Living alone	0.012	–0.027 to 0.002	0.102	0.313	–1.279 to 1.905	0.700
• HT	0.009	–0.006 to o.0.024	0.229	0.016	–1.568 to 1.601	0.984
• Previous TIA	0.009	–0.006 to o.0.024	0.242	–0.013	– 1.611 to 1.584	0.987
• Previous stroke	0.009	–0.006 to o.0.023	0.255	–0.044	–1.619 to 1.530	0.956
• Diabetes mellitus	0.011	–0.004 to 0.026	0.138	0.271	–1.313 to 1.856	0.737
• AF	0.001	–0.005 to 0.025	0.201	0.062	–1.527 to 1.651	0.939
• mRS pre-stroke 0–2	0.015	0.000 to 0.029	0.055	0.398	–1.231 to 2.026	0.362
• NIHSS at admission	–0.038	–0.038 to –0.008	0.003	–2.665	–4.338 to – 0.992	0.002
• Ischaemic stroke	0.008	–0.007 to 0.023	0.284	–0.073	–1.657 to 1.510	0.928
Adjusted for all	0.006	–0.009 to 0.021	0.420	–1.403	–3.039 to 0.234	0.093

Linear regression analyses showed no significant association between attainment of the stroke care pathway goals and self-perceived health at 3 months after stroke using the EQ index (B = –1.40, 95% CI –3.039 to 0.234, *p* = 0.093) and EQ-VAS (B = 0.006, 95% CI –0.009 to 0.021, *p* = 0.42) as outcome variables. This applied both to the unadjusted and adjusted models. However, when adjusting for stroke severity (NIHSS) on admission we found that attaining the goals of the pathway was significantly associated with the EQ index (B = 0.038, 95% CI –0.038 to –0.008, *p* = 0.003) and with the EQ-VAS (B = –2.665, 95% CI –4.338 to –0.992, *p* = 0.002). This indicates that NIHSS might be a suppressor variable.

The mediation analysis showed no mediating effect of thrombolysis upon SPH using the EQ index or EQ-VAS as outcome variables.

## DISCUSSION

In this study our primary objective was to explore the potential association between attaining important goals of a stroke care pathway and self-perceived health 3 months after stroke.

No significant association between attainment of the SCP and the SPH was found using the EQ-5D index and EQ-VAS as outcome variables, and no evident differences in the EQ index and EQ-VAS scores between the attainment and non-attainment groups were identified.

Compared with a non-stroke population using the Danish population norms for the EQ-5D-5L, the EQ5D-5L index was slightly decreased (0.90 vs 0.80), a difference that may have clinical implications according to a study by McClure et al. (23, 26).

Similarly, the EQ-VAS score was reduced compared with a normal population (69.0 vs 77.9) (27).

We found significant differences in demographics between the 2 groups. Patients in the attainment group had better pre-stroke function measured by mRS and were to a lesser degree living alone. Both these factors have previously been shown to facilitate faster recognition of stroke symptoms and reduced time from onset to admission to hospital (28). Additionally, the patients in the attainment group had more severe strokes on admission as measured by NIHSS. Severity of stroke and certain symptoms such as aphasia and paresis are demonstrated to be easier to recognize as a stroke and thereby initiate a faster response.

Previous studies have demonstrated that stroke may have a considerable impact on health-related quality of life (HRQoL) (14, 16). A systematic review found that HRQoL in ischaemic stroke patients was low initially but improved and stabilized by 6 months. However, as in our study using the EQ-5D-index, the utility values were lower than the non-stroke population in the long-term observation. Furthermore, health utility values decreased significantly as stroke severity (mRS) increased (29).

In the same study, the most impaired health dimension assessed by EQ5D was usual activity, such as work, study, or leisure activities. This is in line with the results of our study, where usual activities and pain/discomfort were the most affected dimensions.

When adjusting for stroke severity (NIHSS) on admission we found that attaining the goals of the pathway was significantly associated with the EQ index and the EQ-VAS, which indicates that NIHSS might be a suppressor variable. However, NIHSS hast the largest proportion of missing data (13%) among the potential confounders, and its missingness is informative. Hence, we cannot exclude the possibility that the observed suppressor effect of NIHSS may be due to informative missingness.

This underscores the important role of stroke severity in determining outcomes and highlights the importance of effective pre-hospital and intra-hospital organization to mitigate the consequences of stroke. In our study, we did not have data on rehabilitation that the patients may have received after discharge from the stroke unit. However, as this could impact patients’ SPH, information on rehabilitation measures should be included in future studies.

We were not able to confirm our hypothesis that patients who attained the goals of the SCP had a better SPH than the others, disregarding the effect of stroke severity as a possible suppressor variable.

One explanation might be that most of the included patients exhibited minor stroke symptoms with low mRS (0–2) and low NIHSS scores (0–5), suggesting the stroke’s impact on EQ-5D dimensions was minimal (13). Many of the more severely affected patients were excluded from the study as they were not able to attend a 3-month follow-up or answer the questionnaires. Besides, studies have shown that stroke survivors’ perceptions of health are multifactorial, and that age, comorbidity, educational level, psychosocial factors, and frailty are strongly associated with SPH (9, 30). The study’s timing, just 1 year after stroke care pathway implementation, may have affected the results, particularly regarding how fully the pathway was adopted across regions and hospitals in 2019.

A reduction in pre-hospital time delay causes increased use of revascularization therapies, which in turn results in enhanced functional outcomes (31). More than 5 times as many received thrombolysis in the attainment group (60%) vs the non-attainment group (11%). Research suggests diverse outcomes regarding the impact of thrombolysis on SPH, while thrombectomy is shown to improve SPH at least up to 18 months (32). Further, admission to a stroke unit without delay is proven to improve functional outcome and quality of life. (30) Overall, the reduction of NIHSS from admission to 24 h was substantially higher in the attainment group, 2.0 (5.3 vs 3.3) points compared with the non-attainment group at 0.8 (3.3 vs 2.5) points, and it is reasonable to believe that this is attributable to the high proportion of patients in the attainment group receiving tPA (12). However, these changes are not reflected in the self-perceived health measurements.

Only 22% of the patients achieved 3 or 4 goals of the pathway and qualified for the attainment group. The least achieved SCP measure was contact with the EMS within 15 min from symptom onset. One main reason is probably that knowledge of stroke symptoms is still low or suboptimal. Another explanation that has been demonstrated is that there is an inability to translate knowledge into action, in other words, the patients know the symptoms but are unsure of how to react to them (33). Patients in the attainment group were found to have more severe strokes on admission, and higher NIHSS scores are associated with shorter prehospital delays. Conversely, mild to moderate symptoms might have been underemphasized in public awareness campaigns and in the information provided to patients and caregivers. In evaluating SPH, it is also crucial to consider factors affecting SPH that are similar in people with and without stroke such as, for example, demographic, physical and socioeconomic status, education, and psychological factors (34, 35).

### Strengths and limitations

To the best of our knowledge, our study is the first to evaluate the association between stroke care organization through an SCP designed to minimize delays and improve SPH after stroke.

The major strength of our study was the large sample size. Another strength is that evaluations of the NSR have demonstrated the register’s high quality, and a separate study on inter-rater reliability found that most variables in the register exhibited substantial to excellent reliability (36, 37). However, observational studies inherently carry a risk of selection bias. Consequently, we cannot rule out variations in data quality, which may have influenced the results and limited the study’s generalizability.

Most of the patients included in this study demonstrated minor symptoms with mRS 0–2 and low NIHSS scores, in line with the majority of the Norwegian stroke population (38). Consequently, the findings might be most relevant to this subgroup. In future studies more severely affected patients should be included as well as part 2 of the SCP covering the period from hospital discharge until 3-month follow-up. As this was not fully implemented it was not included in the present study.

One potential limitation in our study is that the included patients had substantially lower NIHSS scores on admission and after 24 h compared with the overall population. Although part of this difference may be explained by higher NIHSS scores among patients who died within 3 months, excluding these patients still revealed a discrepancy. As seen in Table III, the amount of missing data is small for most of the variables listed, but for NIHSS on admission the proportions missing are 523/4,133=13% and for NIHSS after 24 h 935/4,133=23%. This factor may also explain some of the observed discrepancies and could influence the study’s generalizability. Nevertheless, it is important to note that all NIHSS scores fall into the minor stroke range (NIHSS 5.7 and below). Further, the study includes the score of NIHSS, but not the specific symptoms. As aphasia would probably cause difficulties in responding to the EQ-5D questionnaire, we assume that few patients with aphasia were included in the study, which may have affected the results. Another potential criticism is that only 4 goals from SCP are used in the final analyses to evaluate the possible association between SCP and SPH, and the 4 goals we decided to use were known to be important regarding stroke outcome and they had relatively complete data.

We used the EQ-5D-5L as a measure of SPH. As no validated Norwegian dataset is available, we used the Danish dataset and it is unclear to what degree these populations differ. Even though the EQ-5D-5L is a valid measure of SPH, it is a quite simple measure that does not encompass domains like communication and memory impairments. Severely affected stroke patients, particularly those with cognitive or speech impairments, may be unable to complete questionnaires or respond to questions, limiting the value of these scores for these individuals.

Several arguments supporting and opposing VAS as a valuation method exist. The most frequently mentioned arguments, which in some studies have been associated with lower valuations compared with other scales, is that the VAS is not based on choices and that there is no uncertainty involved (39). Another argument is that middle point bias may occur, meaning that the respondents tend to avoid the upper and lower ends of the scale. Conversely, VAS scales are simple, easy to understand, and quick to complete.

It is well known that change in clinical practice takes time. The SCP had only been implemented for 1 year when we conducted this study, which may have affected the results, particularly because the introduction of SCP might have varied between different hospitals and regions. On the other hand, we believe that if the evaluation of the SCP had been postponed for some years, other external factors would have affected the results.

### Conclusion

This study, with a cohort mostly consisting of patients with mild to moderate strokes, assessed the -association between achieving essentials goals of a stroke care pathway aimed at reducing delays in stroke care and self-perceived health after stroke. There was no association between attaining goals of the SCP and SPH.

However, stroke severity may have acted as a suppressor variable, underscoring the importance of stroke severity for SPH. Future studies are needed to explore whether pre- and intrahospital organization in acute stroke care and rehabilitation can impact post-stroke self-perceived health, especially among patients with more severe strokes.

## Footnotes

1 Bergh E, Askim T, Rønning OM, Fjærtoft H, Thommessen B. How achievement of a stroke care pathway is associated with functional outcome after stroke: a national, register-based study. Submitted
